# Potential therapeutic effect of the secretome from human uterine cervical stem cells against both cancer and stromal cells compared with adipose tissue stem cells

**DOI:** 10.18632/oncotarget.2530

**Published:** 2014-09-26

**Authors:** Noemí Eiró, Juan Sendon-Lago, Samuel Seoane, María A. Bermúdez, Maria Luz Lamelas, Tomás Garcia-Caballero, José Schneider, Roman Perez-Fernandez, Francisco J. Vizoso

**Affiliations:** ^1^ Unidad de Investigación, Fundación Hospital de Jove, Gijón, Spain; ^2^ Departamento de Fisiología-CIMUS, Universidad de Santiago de Compostela, Spain; ^3^ Departamento de Ciencias Morfológicas, Universidad de Santiago de Compostela, Spain; ^4^ Universidad Rey Juan Carlos, Facultad de Ciencias de la Salud, Spain; ^5^ Fundación para la Investigación con Células Madre Uterinas (FICEMU), Gijón, Spain

**Keywords:** Mesenchymal stem cell, uterine cervix, breast cancer, tumor stroma, cancer-associated fibroblasts, macrophages

## Abstract

Evidences indicate that tumor development and progression towards a malignant phenotype depend not only on cancer cells themselves, but are also deeply influenced by tumor stroma reactivity. The present study uses mesenchymal stem cells from normal human uterine cervix (hUCESCs), isolated by the minimally invasive method of routine Pap cervical smear, to study their effect on the three main cell types in a tumor: cancer cells, fibroblasts and macrophages. Administration of hUCESCs-conditioned medium (CM) to a highly invasive breast cancer MDA-MB-231 cell line and to human breast tumors with high cell proliferation rates had the effect of reducing cell proliferation, modifying the cell cycle, inducing apoptosis, and decreasing invasion. In a xenograft mouse tumor model, hUCESCs-CM reduced tumor growth and increased overall survival. In cancer-associated fibroblasts, administration of hUCESCs-CM resulted in reduced cell proliferation, greater apoptosis and decreased invasion. In addition, hUCESCs-CM inhibited and reverted macrophage differentiation. The analysis of hUCESCs-CM (fresh and lyophilized) suggests that a complex paracrine signaling network could be implicated in the anti-tumor potential of hUCESCs.

In light of their anti-tumor potential, the easy cell isolation method, and the fact that lyophilization of their CM conserves original properties make hUCESCs good candidates for experimental or clinical applications in anticancer therapy.

## INTRODUCTION

Breast cancer is the most common malignancy in women. Despite early diagnosis, surgery, and adjuvant therapy, a considerable number of patients recur with metastatic disease and die as a result. Tumor initiation is typically conceptualized as the accumulation of genetic and epigenetic mutations in the epithelium that result in recruitment of reactive stroma. Evidence exists indicating that tumor development and progression towards a malignant phenotype depend not only on cancer cells themselves, but also on tumor stroma reactivity [1], which may often dictate tumor outcome in breast cancer [2-5]. The two well-studied cellular components of tumor stroma are cancer associated fibroblasts (CAFs) [6] and cancer-associated macrophages (CAMs) [7]. It has been widely reported that both are key orchestrators of tumor microenvironment, directly affecting neoplastic cell growth, neoangiogenesis, and extracellular remodelling [8]. New therapeutic strategies for breast cancer should consider not only their effect on cancerous cells but also on stromal cells.

Human mesenchymal stem/stromal cells (MSCs) are multipotent cells that possess the capability for self-renewal and are able to differentiate into several types of mesenchymal cells including adipocytes, osteoblasts, and chondrocytes [9-11]. MSCs have been isolated from many types of human adult and fetal tissues, including bone marrow and adipose tissues, umbilical cord, placenta, and the uterus [12-20]. However, this generally has required the use of highly invasive methods. Various studies have shown that properties and paracrine activities of MSCs differ according to their origin [21-22].

In recent years, various studies have focused on the relationship between stem/stromal cells and the oncogenic process [23]. However, to the best of our knowledge, no prior research is available regarding the effect of MSCs secretome (conditioned medium) on the neoplastic growth of both tumor stromal cell types: CAFs and CAMs.

In the present study, we describe the isolation and growth of MSCs from the human uterine cervix (human uterine cervical stromal cells, hUCESCs) using a minimally invasive process. We show that hUCESCs and their secretome, also called conditioned medium (hUCESCs-CM), inhibit the aggressive behavior of cancer cells (cell lines and primary tumors) *in vitro* and reduce tumor growth *in vivo* in a mouse xenograft tumor model. In addition, we report that hUCESCs have an inhibitory effect on CAFs proliferation and invasion, and can inhibit and revert macrophage differentiation.

## RESULTS

### Isolation and characterization of hUCESCs

**Figure 1:**
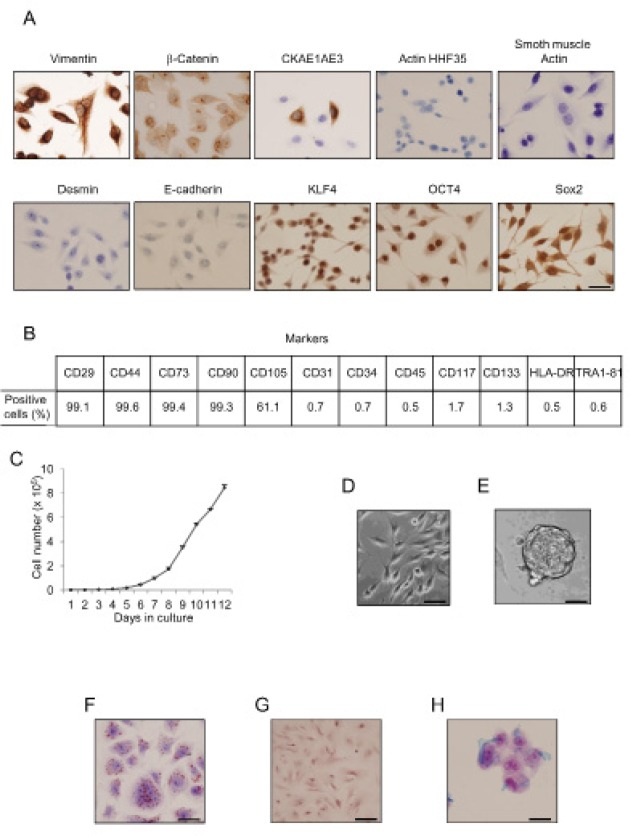
Uterine cervical cells show immune phenotype and functionality of adult MSCs (A): Cells obtained from cervical PAP smears were immnunolabeled with specific antibodies and then evaluated by immunohistochemistry for protein expression. Desmin, actin HHF35, smooth muscle actin, and E-cadherin expression was not detected, while CKAE1AE3 was focally expressed, and vimentin showed strong expression. Specific stem cell markers such as klf4, oct4, and sox2 showed strong immunolabeling in uterine cervical stem cells. Scale bar: 25 μm. (B): Flow cytometry analyses of human uterine cervical stem cells indicated high percentage of CD29, CD44, CD73, CD90 and CD105 proteins, but negative presence of CD31, CD34, CD45, CD117, CD133, HLA-DR, and Tra1-81 proteins. (C): Growth of hUCESCs expressed as number of cells after seeding 2000 cells/well. (D): Normal hUCESCs in culture. Scale bar: 25 μm. (E): hUCESCs form spheroids when cultured in specific medium for 12 days. Scale bar: 100 μm (F): Oil Red O staining as a marker of adipose cells was observed in hUCESCs after 12 days of culture with specific adipose differentiation medium. Scale bar: 25 μm. (G) Alizarin Red S staining as a marker of osteogenic differentiation was observed in hUCESCs after 15 days of culture. Scale bar: 25 μm (H) Alcian Blue staining as a marker of chondrogenic differentiation was observed after 21 days of hUCESCs culture. Scale bar: 25 μm.

hUCESCs obtained from exfoliation PAP smears of the uterine cervix were examined for immunophenotype using immunocytochemistry and flow cytometry, as reported previously by Eiro et al., (World Congress on Cell Science & Stem Cell Research. 2014). hUCESCs are positive for vimentin and β-catenin, and occasional cells also immunostained with pan-cytokeratin antibody (clone AE1AE3) (Figure 1A). In addition, hUCESCs had strong expression of three transcription factors characteristic of embryonic stem cells: OCT4, KLF4, and Sox2. hUCESCs phenotype was also determined by flow cytometry. We found that these cells were positive for CD29, CD44, CD73, CD90 and CD105, while they were negative for CD34, CD45, CD133 (hematopoietic markers), CD31 (endothelial marker), CD117, TRA-1-81 (embryonic stem cell surface marker), and HLA-DR (Figure 1B and supplemental figure 1). This phenotype was observed at different passages. The doubling index of hUCESCs was 1.76 in 24 hours (Figure 1C).

To further evaluate hUCESCs cells, we induced them to form spheroids. After seven days in culture (Figure 1D), individual cells were maintained in suspension culture and at day twelve the cells formed clonal spheroid structures (Figure 1E). We also evaluated the capacity of hUCESCs for differentiation by adding specific culture medium. Adipogenic differentiation was demonstrated by Oil Red O staining (Figure 1F). Calcium deposition, as marker of osteogenic differentiation was evaluated by Alizarin Red S staining (Figure 1G). Finally, secreted extracellular matrix proteoglycans, as markers of chondrogenic differentiation, were observed after Alcian Blue staining (Figure 1H).

### Effect of hUCESCs on proliferation of human breast cancer cells

**Figure 2:**
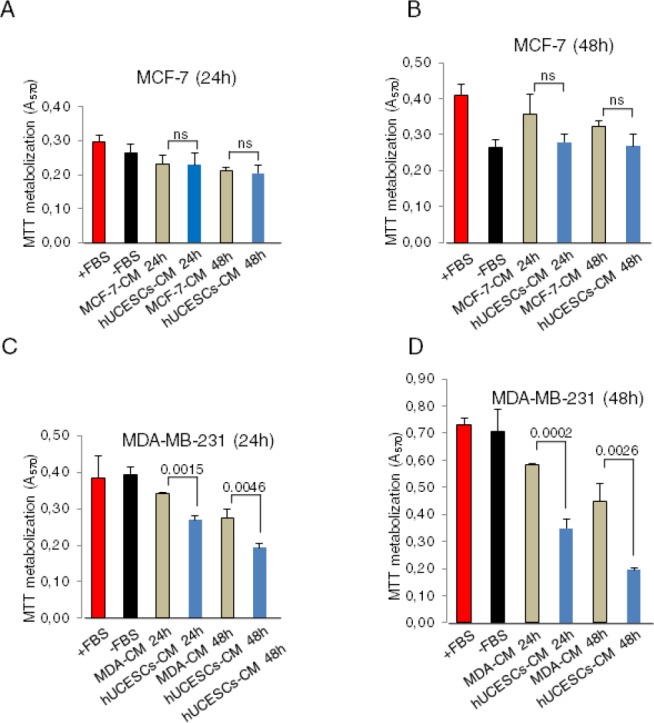
hUCESCs-CM reduced cell proliferation in MDA-MB-231 cells but not in the MCF-7 cells (A-B): MTT assay of MCF-7 cells treated for 24 and 48 hours with complete medium (+FBS), incomplete medium (−FBS), 24 and 48-hour MCF-7-CM, and 24 and 48-hour hUCESCs-CM. (C-D): MTT assay of MDA-MB-231 cells treated as above. ns: not significant.

**Figure 3:**
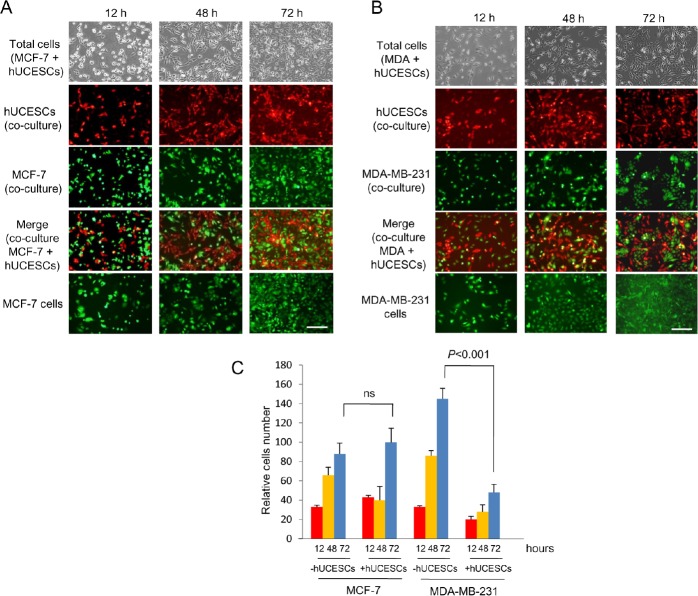
hUCESCs decreased growth of MDA-MB-231 but not MCF-7 cells in co-culture (A): MCF-7 cells (1 × 10^5^) were labeled with CellTracker Green dye and plated in 6-well plates. Four hours later 1 × 10^5^ hUCESCs labeled with CellTracker Red dye were added to MCF-7 cells and co-cultured in incomplete medium (without FBS) for 72 hours. Images were taken at 12, 48 and 72 hours. The last line shows an example of MCF-7 cell growth in incomplete medium (−FBS), which was used as control. Scale bar: 50 μm. (B): MDA-MB-231 cells were labeled and co-cultured with hUCESCs as described in (A) for MCF-7 cells. Scale bar: 50 μm. (C): Relative growth of MCF-7 and MDA-MB-231 cells in co-culture with or without hUCESCs for 12, 48, and 72 hours. ns: not significant.

To explore the possible effect of hUCESCs on breast cancer, after administration of hUCESCs-CM we evaluated the proliferation/cytotoxicity in the non-invasive human breast cancer cell line MCF-7 and in the highly invasive human breast cancer cell line MDA-MB-231. As shown in Figure 2A-B, after 24 and 48 hours of administration of hUCESCs-CM (from 24 or 48 h) to MCF-7 cells no significant decrease of MTT metabolization was observed, as compared to cells treated with medium without FBS, or MCF-7-CM self-produced for 24 or 48 h. However, when the hUCESCs-CM was administered to the MDA-MB-231 cell line, a significant decrease in cell proliferation was seen at 24 and 48 hours (Figure 2C-D). Previously, similar data were obtained using the human cervical cancer HeLa cell line (Supplemental Figure 2). In addition, when the lyophilized hUCESCs-CM was administred to the aggressive MDA-MB-231 cell line, a dose-dependent inhibition of cell proliferation (Figure 2E) was observed. To evaluate whether the effect of hUCESCs-CM on MCF-7 and MDA-MB-231 cell proliferation was modified by co-culture with hUCESCs, we labeled MCF-7 and MDA-MB-231 cells with a green dye, and hUCESCs with a red dye. We found that while MCF-7 cells co-cultured with hUCESCs grew similarly to MCF-7 cells cultured alone (Figure 3A and 3C), co-culture of MDA-MB-231 cells with hUCESCs significantly (*P* < 0.01) reduced the number of MDA-MB-231cells (Figure 3B and 3C).

### hUCESCs-CM modifies cell cycle and induces apoptosis in the MDA-MB-231 cell line

**Figure 4:**
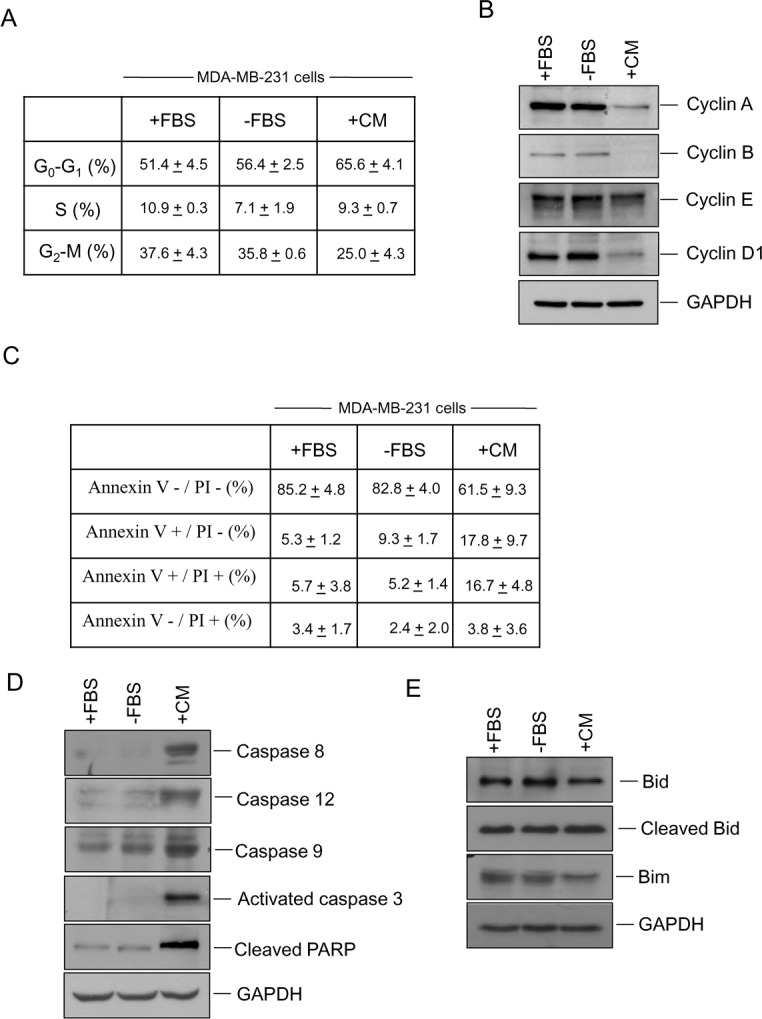
Administration of hUCESCs-CM to MDA-MB-231 cells delayed the cell cycle and increased apoptosis (A): MDA-MB-231 cells were treated for 48 h with DMEM plus 10% FBS (+FBS), incomplete medium (DMEM without FBS, -FBS), or 48-h hUCESCs-CM, and then subject to flow cytometry using propidium iodide (PI). Percentage of cells (mean + standard deviation) in each phase is shown. (B): Western blot of cyclin A, cyclin B, cyclin E, cyclin D1, and GAPDH (used as loading control) of protein extracts from MDA-MB-231 cells treated for 48 h as described in (A). (C): Apoptosis was determined in MDA-MB-231 cells cultured for 48 h with complete (+FBS), incomplete (−FBS), or hUCESCs-CM by flow cytometry using Annexin V/PI. Annexin V+/PI- and Annexin V+/PI+ indicated early and late apoptosis, respectively. (D): Western blot of Caspase 8, -12, -9, activated caspase 3, and cleaved PARP of MDA-MB-231 protein extracts as indicated in (C). (E): Western blots of the anti-apoptotic Bid, cleaved Bid, and Bim proteins in MDA-MB-231 extracts treated as in (C). GAPDH was used as loading control.

Given that hUCESCs-CM significantly decrease proliferation of MDA-MB-231 cells, we next evaluated cell cycle and apoptosis as possible mediators. MDA-MB-231 cells were cultured for 48 h with DMEM plus 10% FBS (+FBS), DMEM without FBS (−FBS), or 48-h hUCESCs-CM, and then we performed flow cytometry using propidium iodide (PI) (to evaluate cell cycle), and annexin V/PI to evaluate apoptosis. In addition, we carried out Western blots to evaluate the expression of proteins involved in both cell cycle and apoptosis. Our results indicate that CM- treated cells significantly increased their G0-G1 phase in relation to cells treated with complete (+FBS, *P* = 0.016) or incomplete (−FBS, *P* = 0.03) medium, and decreased their G2-M phase in relation to +FBS-cultured cells (*P* = 0.023) and –FBS-cultured cells (*P* = 0.013) (Figure 4A). In addition, a visible decrease in cyclin A, cyclin B, and cyclin D1 protein expression was observed in CM-treated cells (Figure 4B). Treatment of MDA-MB-231 cells with hUCESCs-CM induced an increase of Annexin+/PI+ (16.7 + 4.8%) positive cells vs. cells cultured without FBS (−FBS 5.2 + 1.4%) suggesting that hUCESCs-CM induces late apoptosis (Figure 4C). Immunoblots of protein extracts from MDA-MB-231 cells treated with hUCESCs-CM showed a clear increase in caspase-8, -12, -9, activated caspase-3, and cleaved PARP (Figure 4D), and a decrease of Bid and Bim (Figure 4E), with respect to cells treated with complete (+FBS) and incomplete (−FBS) medium.

### Invasion, 3D culture formation, tumor growth, and survival rate are modified by treatment with hUCESCs-CM

**Figure 5:**
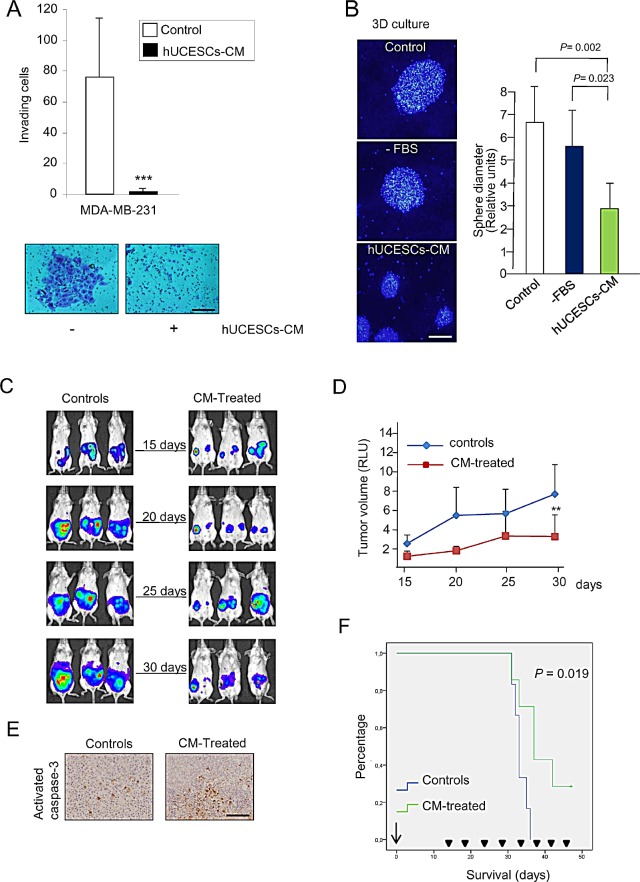
hUCESCs-CM inhibited invasion, 3D growth, and tumor volume in a xenograft mouse model (A): 48-h hUCESCs-CM significantly decreased MDA-MB-231 cell invasion through a matrigel matrix, as compared to controls. Scale bar: 75 μm. (B): Administration of hUCESCs-CM for 10 days significantly reduced 3D growth of MDA-MB-231 cells, as compared to cells treated with complete (+FBS) or incomplete (−FBS) medium. Scale bar: 200 μm. (C): Thirteen SCID mice were injected with MDA-MB-231-luc cells in the mammary fat pad. Fifteen days later, seven mice were intratumorally injected every five days with 150 μl of hUCESCs-CM (CM-treated) and six mice injected with incomplete medium (−FBS, controls). Representative images from controls and CM-treated mice were taken at 15, 20, 25, and 30 days. (D): Tumor volume was determined by measuring luminescence. Values are expressed as mean + standard deviation of relative luminescence levels. **: *P* = 0.011 vs. controls. (E): Immunohistochemical detection of activated caspase-3 expression in representative tumors of SCID mice treated with CM and placebo, as described in (C). Scale bar: 100 μm. (F): Kaplan-Meier plot of overall survival (OS) in CM-treated mice vs. control mice. Mice treated with hUCESCs-CM had a longer OS compared to control mice. The difference was statistically significant (*P* = 0.019).

We explored whether hUCESCs-CM af­fected invasion of MDA-MB-231 cells through a matrigel matrix. Figure 5A shows a significant (*P* < 0.001) decrease in MDA-MB-231 cell invading capacity in presence of hUCESCs-CM as compared with cells in presence of incomplete medium (−FBS). We also explored three-dimensional growth of MDA-MB-231 cells. Treatment with the hUCESCs-CM showed a substantial decrease in sphere diameter, which was not significant when the cells were treated with incomplete medium (CM, mean diameter = 2.8 + 1.0 vs. -FBS, mean diameter = 5.7 + 1.6, arbitrary units, *P* = 0.023) (Figure 5B).

We next evaluated the effect of intratumoral administration of hUCESCs-CM *in vivo* using the severe immunodeficient (SCID) mouse tumor xenograft model. Mice were injected with MDA-MB-231 cells stably transfected with the luciferase vector in the mammary fat pad. 15 days later, when the tumor became visible, mice were injected intratumorally every 5 days with either incomplete medium (controls) or hUCESCs-CM (150 μl), and monitored externally by luminescence (Figure 5C). A significant decrease (*P* = 0.011) in tumor volume was observed after 15 days of treatment with hUCESCs-CM (at day 30) (Figure 5D). On day 33, two animals (one control and one CM-treated mouse) were euthanized, their tumors removed, and analyzed by immunohistochemistry for activated caspase-3 (as indicator of apoptosis). Figure 5E shows a significantly higher activated caspase-3 expression in the hUCESCs-CM-treated mouse as compared to the control mouse. To evaluate mouse survival rate, the remaining mice were injected every 5 days either with hUCESCs-CM or with incomplete medium, and observed until day 47. Kaplan-Meier survival plots (Figure 5F) indicate that mice treated with hUCESCs-CM had a longer overall survival (*P* = 0.019).

### hUCESCs-CM reduces proliferation in breast tumors with high proliferation rate

**Figure 6:**
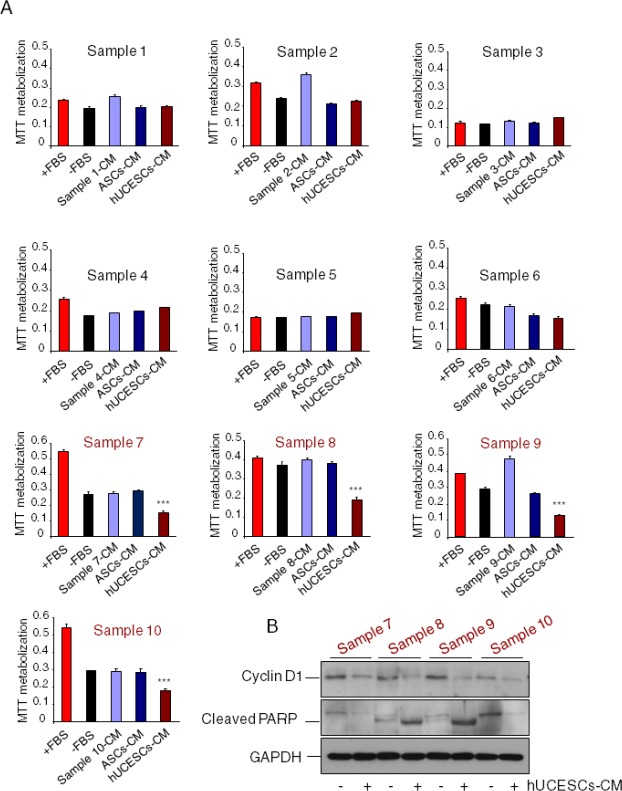
Cell proliferation in primary cultures from human breast tumors with high proliferating rate was significantly reduced after administration of hUCESCs-CM (A): Ten primary cultures from human breast tumors were treated with: a) complete medium (+FBS), b) incomplete medium (−FBS), c) conditioned medium (CM) self-produced for 48 h, d) CM produced for 48 h by adipose-derived stromal cells (ASCs-CM), and e) hUCESCs-CM produced for 48 h. After 48 hours of culture, an MTT assay was carried out to evaluate cell proliferation. Cultures with high proliferation rate (sample 7 to 10, in red) showed a significantly (***: *P* < 0.001) decreased proliferation after treatment with hUCESCs-CM, as compared to others treatments. (B): Protein extracts from primary cultures with high proliferating rate treated with hUCESCs-CM or with incomplete medium (−FBS) were incubated with cyclin D1, cleaved PARP, and GAPDH (used as loading control) antibodies and assayed for Western blot.

Our next step was to evaluate the effect of administration of hUCESCs-CM on primary cultures of human breast tumors. Ten primary cultures were analyzed for cell proliferation using the MTT assay. Similarly to either incomplete medium (−FBS), self-produced CM or ASCs-CM, administration of hUCESCs-CM had no significant effect on cell proliferation on breast tumor cells with a low proliferation rate (sample 1 to 6). On the other hand, administration of hUCESCs-CM induced a significant (*P* < 0.001) decrease in cell proliferation (Figure 6A) in primary cultures with a high proliferation rate (sample 7 to 10). A clear decrease in Cyclin D1 and increased cleavage of PARP protein expression was observed in high proliferative hUCESCs-CM treated cells (Figure 6B).

### Cancer associated fibroblasts are a target of hUCESCs

**Figure 7:**
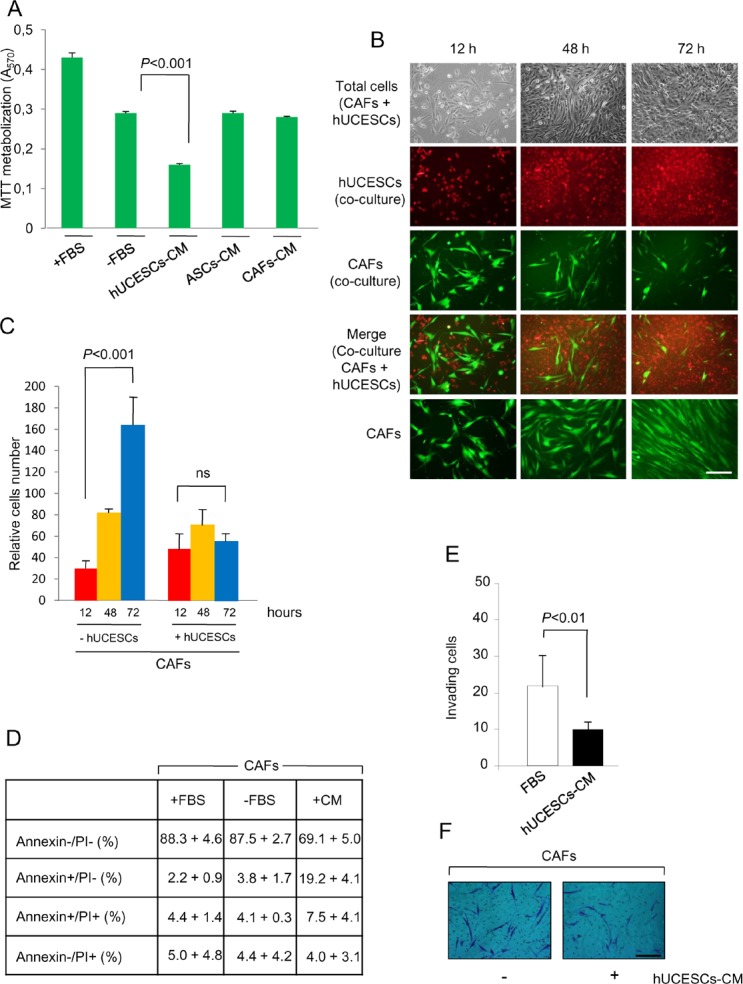
Proliferation, apoptosis and invasion of CAFs was modified after hUCESCs-CM treatment (A): CAFs were obtained from human breast tumors as described in material and methods section and treated with: a) complete medium (+FBS), b) incomplete medium (−FBS), c) conditioned medium (CM) self-produced for 48 h, d) CM produced for 48 h by adipose-derived stromal cells (ASCs-CM), and e) hUCESCs-CM produced for 48 h. After 48 hours of culture, an MTT assay was carried out to evaluate cell proliferation. A significant (*P* < 0.001) decrease in CAFs proliferation after treatment with hUCESCs-CM was observed, as compared to others treatments. (B): CAFs (1 × 10^5^) were labeled with CellTracker Green dye and plated in 6-well plates. Four hours later 1 × 10^5^ hUCESCs labeled with CellTracker Red dye were added to CAFs and co-cultured in incomplete medium (without FBS) for 72 hours. Images were taken at 12, 48 and 72 h. The last line shows an example of CAFs growth in incomplete medium (−FBS), which was used as control. Scale bar: 40 μm. (C): Relative growth of CAFs co-cultured with or without hUCESCs during 12, 48 and 72 h. The cells were labeled and grown as described in (B). (D): Apoptosis was determined in CAFs cultured for 48 h with complete (+FBS), incomplete (−FBS), or hUCESCs-CM by flow cytometry using Annexin V/Propidium Iodide (PI). Annexin V+/PI- and Annexin V+/PI+ indicated early and late apoptosis, respectively. (E): 48-h hUCESCs-CM significantly decreased CAFs invasion through a matrigel matrix. (F): Representative example of CAFs invasion, as described in (E). Scale bar: 100 μm.

Given that cancer associated fibroblasts (CAFs) are involved in the tumorgenicity, we isolated CAFs from one breast tumor with a high proliferation rate, and evaluated the effect of hUCESCs-CM administration to them. Figure 7A shows a significant (*P* < 0.001) decrease in CAFs proliferation after treatment with hUCESCs-CM, but not after treatment with ASCs-CM or CAFs-CM. Co-culture of CAFs with hUCESCs for 72 hours also significantly (*P* < 0.001) reduced number of CAFs as compared to growth of CAFs alone (Figure 7B and 7C). CAFs apoptosis was evaluated 48 hours after treatment either with FBS, without FBS, or with hUCESCs-CM (Figure 7D). We observed a significant (*P* < 0.001) increase in early apoptosis (Annexin+/PI-) in CAFs treated with hUCESCs-CM (19.2 + 4.1%) as compared to CAFs treated with medium plus FBS (2.2 + 0.9%) and medium without FBS (3.8 + 1.7%). Invasion of CAFs was also significantly reduced in presence of hUCESCs-CM with respect to FBS alone (Figure 7E and 7F).

### Inhibition and reversion of monocyte to macrophage differentiation by hUCESCs-CM

**Figure 8:**
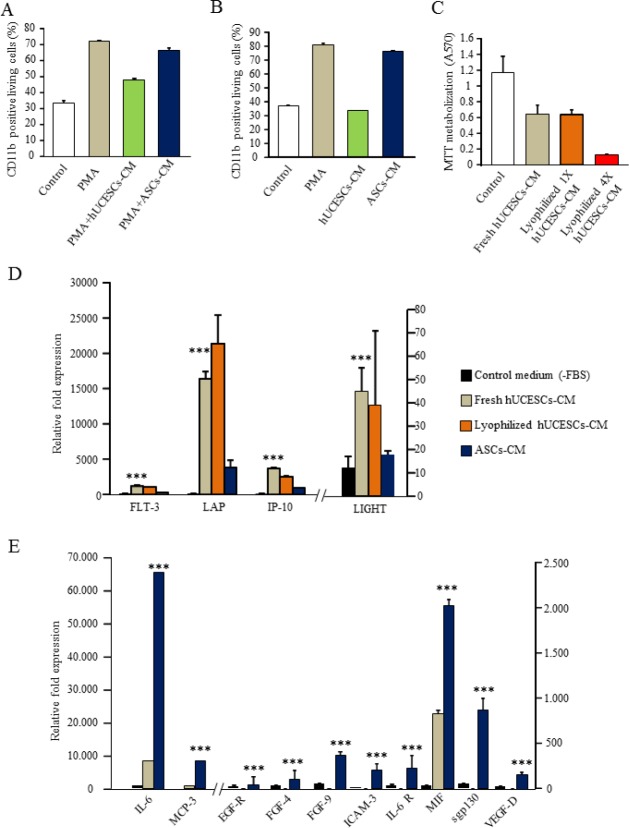
(A): Inhibition of macrophages differentiation with PMA stimulation in presence of hUCESCs or ASCs conditioned medium (B): Reversion of macrophages differentiation: PMA stimulation during 24h and then addition of hUCESCs or ASCs conditioned medium for another 24 hours. (C): MTT assay of MDA-MB-231 cells treated with fresh and lyophilized hUCESCs-CM. Lyophilized hUCESCs-CM was tested at 1X and 4X. (D): High cytokine expression in 48h-CM from hUCESCs (fresh and lyophilized) compared with 48h-CM from ASCs and control media. (E): High cytokine expression in 48h-CM from ASCs compared with 48h-CM from hUCESCs and control media. Bars represents mean ± SD of signal intensity value of each cytokine in hUCESCs-CM, ASCs-CM and control media as detected by RayBio human cytokine antibody array. ***represents p < 0.001 vs control medium and ASCs-CM.

The inhibition of monocyte to macrophage differentiation was performed with PMA treatment during 24 hours in presence of conditioned medium from hUCESCs or from ASCs (StemPro^®^, Invitrogen) or control medium. As shown in Figure 8A, the basal level of expression of CD11b was 34%, and compared with the PMA treated control U937 cells, the percentage of positive cells for CD11b decreased from 73% in PMA treated U937 cells to 48% in hUCESCs-CM treated U937 cells. The percentage of CD11b expression for U937 cells treated with ASCs-CM was 67%. These data indicate an inhibition or protection of macrophage differentiation in the presence of hUCESCs-CM. In Figure 8B, the reversion of macrophage differentiation is shown: PMA stimulation during 24 hours followed by addition of hUCESCs-CM for another 24 hours. Basal level of U937 CD11b expression was 38% and compared with the PMA treated control U937 cells, the percentage of CD11b positive cells decreased from 82% in PMA treated U937 cells to 34% in U937 cells treated with hUCESCs-CM. Nevertheless, the CD11b expression in ASCs-CM treated U937 cells was 77%. It is worth noting that the viability of the U937 cells, in all conditions and both tests, was higher than 80% (data not shown). These data indicate that hUCESCs-CM can reverse macrophage differentiation demonstrated by the baseline expression of CD11b.

### hUCESCs produces cytokines involved in anti-tumor effect

Using a human cytokine antibody array we evaluated factors potentially involved in the anti-tumor effect of hUCESCs-CM. In addition, with the aim to facilitate the use of hUCESCs-CM in future experimental and clinical researches, we have lyophilized it. Lyophilized hUCESCs-CM showed the same activity than fresh hUCESCs-CM (Figure 8C), and of the 174 cytokines analyzed, 4 cytokines seem to be relevant, due to their higher expression in hUCESCs-CM, in both fresh and lyophilized form, and their known role in tumor inhibition. Indeed, we observed at least 2.5 fold increase in the level of TNFSF14 (LIGHT) factor in hUCESCs-CM compared to ASCs-CM (45 vs 18, p<0.001); whereas FLT-3 ligand, IP-10 (or CXCL-10) and LAP are increased at least 3.5 to 4 fold, (FLT-3 ligand: 1146 vs 315, p<0.001; IP-10: 3623 vs 909 p<0.001, and LAP: 16380 vs 3840, p<0.001), in hUCESCs-CM compared to ASCs-CM (Figure 8D). In addition, ASCs-CM presents higher expression of epidermal growth factor (EGFR), fibroblast growth factor (FGF) 4 and 9, intercellular adhesion molecule 3 (ICAM3), interleukine (IL) –6 and its receptor (IL6R), monocyte-specific chemokine 3 (MCP3, also called CCL7), migration inhibitory factor (MIF), soluble glycoprotein 130 (sgp130), vascular endothelial growth factor D (VEGFD). These markers of tumor progression were not detected or comparatively were at lower levels in the hUCESCs-CM. (Figure 8E).

## DISCUSSION

In this study, MSCs were isolated for the first time from the normal human uterine cervix using a minimally invasive procedure (routine PAP smear). These cells were evaluated for their effect on cancer cell lines, primary cultures and stromal cells *in vitro* and in a mouse xenograft tumor model *in vivo*.

According to the minimal criteria proposed by the “Mesenchymal and Tissue Stem Cell Committee of the International Society for Cellular Therapy” [24], we found that hUCESCs differentiated into adipocytes, osteocytes and chondrocytes. In addition, we found that hUCESCs expressed several markers described for MSCs and did not express hematopoietic markers, as decribed previously [20]. However, important differences between hUCESCs and other MSCs should be highlighted. The method for obtaining hUCESCs (Pap cervical smear) is much less invasive and painful that those used to obtain cervical MSCs (biopsy or hysterectomy) or other MSCs (from the bone marrow or adipose tissue). In addition, hUCESCs can be isolated in high quantities, and have a high proliferative rate, making it possible to quickly obtain a huge amount of stem cells or conditioned medium for research and clinical use.

With respect to the role of adult stem cells in tumorigenesis, contradictory results have been described regarding induced pro- or anti-tumor effects, both *in vitro* and *in vivo* [23]. These discrepancies in MSC functionality may also be due to their heterogeneity and to the fact that no specific surface markers currently exist to isolate a more homogeneous population. Therefore, a possible alternative would be to find a MSC-tissue source providing a unique anti-tumor activity. Our results show specific anti-tumor effects of hUCESCs-CM on proliferation, apoptosis, and tumor-cell invasiveness, which differ from those reported for other MSCs [25-27]. Given the fact that tumor stroma plays a fundamental role in tumor growth, invasion and dissemination and that CAFs are the prevailing component of tumor stroma [28-30], we explored the effect of hUCESCs-CM treatment on CAFs [31]. We found that co-culture with hUCESCs as well as treatment with hUCESCs-CM significantly reduced CAFs cell proliferation and invasion, and increased early apoptosis. This finding could be of great importance in light of the fact that CAFs promote tumor onset and progression in a variety of ways (angiogenesis, invasion, and metastasis) as well as mediating drug resistance. Indeed, CAFs are considered a therapeutic target in current clinical trials [32]. Another key cellular component of tumor microenvironment [7] are CAMs. They are considered the most powerful inhibitors of anti-tumor immunity and the greastest barrier to successful immunotherapy [33]. Therefore, we explored the effect of hUCESCs-CM treatment on monocyte to macrophage differentiation. We observed that treatment with hUCESCs-CM significantly inhibited and reversed macrophage differentiation. It has been well documented that high infiltration of CAMs directly correlates with a poor prognosis in solid tumors, including breast cancer [34-36].

Our study revealed that hUCESCs-CM had a higher concentration than control media or ASCs-CM of factors such as LIGHT (or TNFSF14), Fms-related tyrosine kinase 3 ligand (FLT-3 ligand), interferon-gamma-inducible protein-10 (IP-10) and latency-associated protein [24]. These factors have been associated with induction of apoptosis, inhibition of cell growth, reduced cell invasion, and tumor inhibition. LIGHT induces apoptosis through the inactivation of c-IAP1 (apoptosis inhibitor 1), leading to activation of caspase-9, -7, and –3 [37]. Furthermore, it has been described that FLT-3 ligand inhibits tumor growth [38] and that IP-10 has anti-tumor effects through angiostatic and immunogenic action [39]. Indeed, IP-10 is a M1- associated chemokine, reported to have a high tumoricidal capacity [40]. It has also been described that IP-10 attenuates fibroblast accumulation [41]. Therefore, this factor could at least partly explain the effect of hUCESCs-CM on stromal cells. Regarding LAP, it is well documented to interact directly with transforming growth factor beta (TGFb) activators, thus activating TGFb, which in turn acts as a potent growth inhibitor [42]. It is also noteworthy that, compared to ASCs-CM, hUCESCs-CM contain lower levels of factors known to participate in cancer progression, such as EGFR [43], FGF 4 and 9 [44-45], ICAM3 [46], IL6 [47], IL6R [48], MCP3 (CCL7) [49], MIF [50], sgp130[48] and VEGFD[51]. Thus, indicating that the properties of MSCs and their secretomes vary depending on source. All of these findings suggest that a complex paracrine signaling network is implicated in the anti-tumor potential of hUCESCs.

It is not surprising that the human cervix might be a reservoir of MSCs with precise site-specific functions. In fact, the cervical transformation zone is a critical playground, where fundamental biological events take place, some of which may be crucial for the host's wellbeing. One epithelium, i.e., monolayer glandular endocervical epithelium, is constantly transformed into another, multilayer stratified exocervical epithelium, which is more resistant against external agents, presumably due to the pool of underlying MSCs. This constant process of so-called squamous metaplasia especially favors the accumulation of mutations and the eventual development of a malignant phenotype, particularly when viruses, such as the human papilloma virus (HPV), are involved [52]. Furthermore, regeneration and inflammation are closely linked in this setting and constitute a standard model of carcinogenesis [53]. It is therefore reasonable to hypothesize that MSCs are in possession of some intrinsic defense mechanisms against inherent vulnerability from malignant transformation that would otherwise occur much more frequently. It could be that hUCESCs act as “guardians of their environment” by preventing adult cells from acquiring a malignant phenotype, through modulation of their proliferation rate and induction of apoptosis if they become dangerous for the host. Indirectly, our results clearly support this, as hUCESCs were found to exert no action on low-proliferating epithelial cancer cells (the MCF7 cell line), whereas their anti-tumor activity against highly proliferating and metastasis-producing cancer cells (the MDA-MB-231 cell line) was striking.

In summary, MSCs were isolated from the normal human uterine cervix by means of routine Pap cervical smears for the first time. Our findings provide evidence that administration of hUCESCs-CM to the highly invasive breast cancer MDA-MB-231 cell line and to human breast tumors with high cell proliferation rates reduces cell proliferation, modifies the cell cycle, induces apoptosis, and decreases invasion. In addition, in a xenograft mouse tumor model, intratumoral administration of hUCESCs-CM reduces tumor growth and increases overall survival. Importantly, hUCESCs-CM treatment to CAFs from tumor stroma also reduces cell proliferation, induces apoptosis and decreases invasion. It is also noteworthy that hUCESCs-CM inhibit and reverse macrophage differentiation. These effects could be at least partly explained by the presence of factors such as LIGHT, FLT-3 ligand, IP-10 and LAP in hUCESCs-CM, and the absence or lower levels of several factors related to cancer progression.

Considering their anti-tumor capabilities, the easy method for isolating and cultivating cells and the fact that lyophilizing their CM conserves properties, hUCESCs may have the potential to provide us with future anticancer therapies.

## METHODS

### Ethics statement

All human specimens were encoded to protect patient confidentiality and processed under protocols approved by our Regional Research Committee. Breast cancer tissues and cervical smears were obtained from patients who underwent surgery or a routine gynecological check-up at Fundación Hospital de Jove, Asturias, Spain. All patients provided informed written consent.

### Isolation and growth of hUCESCs and cell cultures

hUCESCs were obtained from routine Pap cervical smears performed on non-menstruating caucasian women. In all cases, results of the routine Pap from these fertile women showed no abnormalities. Briefly, the cytological sample was enzymatically disaggregated. Then, the sample was centrifuged 5 min at 400 g and the pellet was collected and seeded in a culture plate. The sample was cultured in DMEM-F12 with glutamine, penicillin and streptomycin, 10% FBS, epidermal growth factor (EGF, Gibco, Life Technologies, Paisley, UK), hydrocortisone (Sigma-Aldrich, St. Louis, MO, USA), insulin (Gibco, Life Technologies), in an air-CO_2_ (95:5) atmosphere at 37ºC. The subculture of cells was carried out with trypsin as described below. The human cervical cancer (HeLa), breast adenocarcinoma cell lines MCF-7 and MDA-MB-231 and monocyte cell line U937 were obtained from the European Collection of Cell Cultures (Salisbury, UK). The initial experiment to test the interaction of hUCESCs with tumor cells was carried out with a site-specific, cervical cancer cell line (HeLa). However, once proven that such an interaction existed, we decided to carry out the rest of experiments with breast cancer tumor lines, both commercially available and established at our own laboratory in order to avoid the known cross-contamination problems associated with HeLa cells, which often result in the contamination of any other cell lines used in a given laboratory [54]. Cell lines were grown in 90-mm Petri dishes in DMEM or RPMI-1640 (U937 cells) supplemented with 10% FBS, penicillin and streptomycin in an air-CO_2_ (95:5) atmosphere at 37ºC. Confluent cells were washed twice with phosphate-buffered saline and harvested by a brief incubation with trypsin-EDTA solution (Sigma-Aldrich) in PBS. Ten primary cultures from women with breast tumors were used in the present study. Cancer-associated fibroblasts (CAFs) were obtained as follows: after tumor resection, the pathologist examined and obtained a representative piece of tumor tissue. The tumor was mechanically disaggregated by mincing with scalpel and scissors to 1–2 mm^3^ in a 6 well plate. Tissue was digested with 1.25 mg/ml of collagenase A in complete-DMEM-F12 medium for 48 hours in an air-CO_2_ (95:5) atmosphere at 37ºC. For 7 days, primary cultures were grown in a DMEM-F12 medium, supplemented with 10% FBS, 100 U/ml penicillin, and 100 μg/ml streptomycin, 10 ng/ml epidermal growth factor (EGF), 0.5 μg/ml hydrocortisone and 10 μg/ml insulin. Afterwards, cells were cultured, at 1500-2000 cells/cm^2^, in a DMEM-F12 medium supplemented with 10% FBS, 100 U/ml penicillin, and 100 μg/ml streptomycin. At passage 4, mostly fibroblasts remained, then the purity of CAFs was assessed by immunohistochemistry (positive expression of α-smooth muscle actin and vimentin, and negative expression of desmin), and flow cytometry (at least 70% of CD90 clon AS02 positive cells).

hUCESCs, primary cultures from human breast tumors, CAFs, and human adipose tissue-derived mesenchymal stem cells (ASCs, StemPro^®^, Invitrogen, Life Technologies) were grown, at 1500-2000 cells/ cm^2^, in 90-mm Petri dishes in DMEM-F12 (1:1) supplemented with 10% FBS, 100 U/ml penicillin, and 100 μg/ml streptomycin in an air-CO2 (95:5) atmosphere at 37ºC.

Conditioned medium (CM) from hUCESCs, ASCs, CAFs, MCF-7, and MDA-MB-231 cells was obtained by culturing the cells to 70% confluence as described above. Afterwards, the cells were washed three times in PBS, and cultured again in DMEM-F12 without FBS. After 24 or 48h, the medium was centrifuged for 5 min at 300g, the supernatant was collected, and used immediately.

hUCESCs-CM was also lyophilized (SP Scientific, 25L Genesis 5 Q EL-85, Gardiner, NY, United States) and then store at −80ºC until used. The lyophilized powder was then resuspended just before used in ddH_2_O.

For spheroid formation, hUCESCs were cultured in DMEM/F12 medium (1:1) (Invitrogen), 1% B27 (Invitrogen), 10 ng/ml EGF, 5 ng/ml fibroblast growth factor 2 (FGF-2), 100 IU/ml penicillin, and 100 μg/ml streptomycin in a 60 mm dish, and 12 days later the spheroids were photographed

To induce adipose differentiation, hUCESCs were cultured in human MSC Differentiation Bullet kit-Adipogenic medium (Lonza Biologics, Walkersville, USA) in a 60 mm dish during 12 days and then formaldehyde-fixed for Oil Red O staining. Osteogenic differentiation was performed with the StemPro^®^ osteogenic differentiation medium (Gibco, Grand Island, NY, USA) with Osteogenesis Suplement during 15 days, and differentiation was assessed by Alizarin Red S staining (Sigma). For chondrogenic differentiation, cells were cultured during 21 days with the differentiation media StemPro^®^ (Gibco) with Chondrogenic Suplement (Gibco). Differentiation was evaluated by Alcian Blue staining.

For co-culture assays, the cells were cultured as described above. The medium was removed at 70% confluence and the cells were labelled with pre-warmed CellTracker^™^ solution (MCF-7, MDA-MB-231, and CAFs with CellTracker^™^ GREEN CMFDA, and the hUCESCs with CellTracker^TM^ RED CMPTX; Invitrogen, Eugene, USA) as per the manufacturer's instructions. Then, 1 × 10^5^ MCF-7, MDA-MB-231, or CAFs cells/well were plated in 6-well plates, and four hours later 1 × 10^5^ hUCESCs cells were added to the MCF-7, MDA-MB-231, or CAFs and co-cultured for 72 h. Images were randomly photographed at 12, 48 and 72 hours with a high-resolution digital camera (Olympus DP 72; Olympus Corp., Tokyo, Japan). A counting frame (102 μm^2^) was superimposed on the captured image in at least four different fields. Only clearly visible cells were counted in each of the fields using the ImageJ software (National Institutes of Health, Bethesda, MD, USA). Results are expressed as relative number of cells.

Three-dimensional cell culture was performed as previously described [55]. Briefly, a single-cell suspension containing 5 × 10^3^ MDA-MB-231 cells per 100 μL volume of medium, supplemented with 2% (1:1) of Matrigel, was care­fully placed on top of the solidified Matrigel. The culture slides were then placed in six-well plates, 500 μL of medium was added per well, and the cells were cultured for 10 days. MDA-MB-231 cells were then treated with different media: a) DMEM-F12 with 10% FBS (+FBS), b) DMEM-F12 without FBS (−FBS), or c) 48 h-conditioned medium from hUCESCs (hUCESCs-CM) for 1 week. Phase contrast photographs of cells as monolayers, or in three-dimensional cultures, were taken with an Olympus DP72 camera. The quantitation of sphere diameter was performed manually by tracing a straight line across the diameter of the sphere and scoring its value as arbitrary length units.

### Flow cytometry characterization

hUCESCs were stained with a panel of specific monoclonal antibodies: CD29- PE, CD45-FITC, CD90-PE, CD105-PE, HLA-DR-PE (Beckman Coulter, Marseille, France), CD44-PE, CD73-PE, CD31-PE, TRA1-81-FITC (Becton Dickinson, Biosciences Pharmingen, San Diego, CA, USA), CD34-FITC, CD117-PE and CD133-PE (Miltenyi Biotec, Bergisch Gladbach, Germany). 7-amino-actinomycin-D (7-AAD) (Sigma-Aldrich) was added for dead cell discrimination.

Differentiation to macrophages on U937 cell line was monitored by the expression of monocyte differentiation marker CD11b using flow cytometry analysis. Cells were stained with PE-CD11b monoclonal antibody and with 7-AAD to assess cell viability. The Mac-1 (CD11b) antigen is a cell surface marker for macrophages.

Immunophenotyping was performed on the same cell population aliquoted equally into two different tubes. Stained cells were re-suspended in PBS, and analyzed using a Cytomics FC500 flow cytometer (Beckman Coulter). The computed data were analyzed using CXP software provided by the manufacturer.

### Cell proliferation assay

The proliferation rate of hUCESCs was determined by counting the total number of cells in triplicate every day for 12 days. Initially, the cells were seeded at 2000 cells/well in a 6-well plate culture. The doubling time [47] was determined by using the formula: Dt = (**t - t**_0_) log2/(log**N** - log**N**_0_), where **t** and **t**_0_ are the times at which the cells were counted, and **N** and **N**_0_ are the cell numbers at times **t and t**_0_, respectively.

Cell viability/proliferation experiments were carried out using 3-(4,5-dimethylthiazol-2-yl)-2,5-diphenyltetrazolium bromide (MTT) assays as previously described [56]. MCF-7, MDA-MB-231, primary cultures from human breast tumors, or CAFs were plated at a 3 × 10^4^ cells per well in 24-well plates. Twenty-four hours later, the cells were treated with equal volumes (500 μl) of DMEM-F12 with 10% FBS (+FBS), DMEM-F12 without FBS (−FBS), and 24 or 48 h-conditioned medium from MCF-7, MDA-MB-231, hUCESCs (fresh and lyophilized), ASCs, CAFs, or primary cultures of breast cancer tumors, for 24 or 48 h. Absorbance of samples was measured at 570 nm using a multiwell plate reader (Tecan ULTRA Evolution, Männedorf, Switzerland). Results were plotted as the mean + SD values of quadruplicates from at least three independent experiments.

### Western blot analysis

MCF-7, MDA-MB-231 cells, and primary cultures from human breast tumors were lysed at 4ºC in 300 μl of lysis buffer (50 mM HEPES, pH 7.5; 150 mM NaCl; 5 mM EGTA; 1.5 mM MgCl_2_; 1% SDS; 10% glycerol; 1% Triton X-100; 10 mM sodium orthovanadate; 4 mM PMSF, and 50 μg/ml aprotinin). The cell lysate was then centrifuged at 14,000*g* for 5 min at 4ºC, the resulting supernatant was collected, and protein concentration determined by the Bradford method. Western blotting was carried out as described elsewhere [57]. Briefly, 60 μg of total protein was subjected to SDS-PAGE electrophoresis. Proteins were transferred to a nitrocellulose membrane, blocked, and immunolabeled overnight at 4ºC with a primary antibody (see suplemental data table 1), washed three times with PBS-Tween-20, and incubated with the appropriate secondary antibody for 1 hour. The signal was detected with the Pierce ECL Western blotting substrate (Thermo Scientific, Rockford, IL, USA), and visualized by placing the blot in contact with standard X-ray film.

### Cell cycle and Apoptosis assays

Cell cycle and apoptosis assays were carried out by using a Guava flow cytometer (Millipore Corporation, Billerica, MA, USA). Briefly, 2 × 10^5^ cells/well were cultured in: a) DMEM-F12 (1:1) supplemented with 10% FBS, b) DMEM-F12 (1:1) without FBS, and c) hUCESCs-CM. Forty eight hours later, cells were harvested, fixed with 70% cold ethanol for 30 minutes, washed with PBS, and incubated with ribonuclease (100 μg/ml), and propidium iodide (PI, 50 μg/ml) for 60 minutes in darkness, for cell cycle evaluation. Apoptosis analyses were performed using Annexin V-FITC. Cells (2 × 10^5^) were harvested, washed twice with PBS, and resuspended in 1X binding buffer (10 mM Hepes (pH 7.4), 140 mM NaCl, and 2.5 mM CaCl_2_). 5 μl of FITC-Annexin V was added and incubated for 15 min at room temperature in darkness. Finally, 400 μl of 1X binding buffer was added to each tube, and analyzed.

### Cell Invasion Assay

Assays were performed in BD BioCoatMatrigel invasion chambers according to the manufacturer's instructions (BD Biosciences, Madrid, Spain). Filters precoated with Matrigel were used for examining cell invasion. MDA-MB-231 cells or CAFs were placed into the upper chamber in 0.5 ml of DMEM serum-free medium (5 × 10^4^ cells per filter). DMEN-F12 (1:1) culture medium supplemented with 20% FBS (controls) or hUCESCs-CM from 48-h culture, was placed in the lower chamber supplemented with 20% FBS. After incubation for 22 hours, cells that had migrated to the lower surface of the filters were fixed in methanol for 2 minutes at room temperature, stained using crystal violet for 2 minutes, visualized and counted. Values for cell migration or invasion were expressed as the mean number of cells per microscopic field over four fields per one filter for duplicate experiments.

### Animal studies

All animal studies were approved by the University of Santiago de Compostela Ethics Committee for Animal Experiments. Age-matched female mice between 6-8 weeks old, homozygous for the severe combined immune deficiency spontaneous mutation (CB17-Prkdc^scid^, named SCID, Parc Recerca Biomedica, Barcelona, Spain) were used for xenografting studies. Thirteen SCID mice (6 controls and 7 treated) were injected subcutaneously with 3 × 10^6^ MDA-MB-231 cells stably transfected with the pcDNA3-luciferase vector (MDA-MB-231-luc cells) into the left and right flanks. Fifteen days after cell injection, the mice were injected intratumorally (150 μl) with 48-hours hUCESCs-CM or with placebo every five days until day forty-seven. After luciferin injection (150 mg/kg), tumor growth was monitored externally by luminescence using the *In Vivo* Imaging System (IVIS, Caliper Life Sciences, Alameda, CA, USA). An intensity map was obtained using the Living Image software (Caliper Life Sciences). The software uses a color-based scale to represent the intensity of each pixel (from blue representing low to red representing high). One control and one CM-treated mouse were sacrificed at day thirty-one, and tumors excised, fixed in 10% neutral buffered formalin for 24 hours and embedded in paraffin for histological and immunohistochemistry studies. All remaining mice were monitored for survival analyses.

### Immunocytochemistry

hUCESCs were cultured as described above. 3 × 10^4^ cells were seeded in slides, and fixed for 10 minutes in 96% ethanol, before processing for immunocytochemistry. Mouse tumors were immersion-fixed in 10% neutral buffered formalin for 24 hours and routinely embedded in paraffin. 4 μm thick sections were mounted on Flex IHC microscope slides (Dako, Glostrup, Denmark). The immunohistochemical (IHC) techniques were automatically performed in an AutostainerLink 48 (Dako). FLEX ready-to-use Dako primary antibodies to CK (clone AE1/AE3), E-cadherin (clone NCH-38), vimentin (clone V9), desmin (clone D33), actin (clone HHF35), smooth muscle actin (clone 1A4), β-catenin (clone beta-catenin-1) were employed. KLF4, OCT4, and Sox2 primary antibodies were obtained from Santa Cruz Biotechnology (Dallas, USA), Millipore, and Sigma-Aldrich, respectively. Epitope retrieval was performed in a PT Link (for 20 minutes at 97ºC) using EnVision FLEX target retrieval solution (pH 9). All antibodies were incubated for 20 minutes at RT. As detection system we used EnVision FLEX/HRP Dako (dextran polymer conjugated with horseradish peroxidase and affinity-isolated goat anti-mouse and anti-rabbit immunoglobulins) for 20 minutes. For E-cadherin, a mouse linker (Dako) was added. Quantitation of immunopositive cells for active caspase-3 expression was performed as previously described [57].

### Monocyte to macrophage differentation

U937 cell line was used for this experiment. Cells were plated in 24-well plates at a density of 1.5 x10^5^ cells/well in DMEM:F12 with 10% FBS and antibiotics. 2ng/mL of phorbol 12- myristate 13-acetate (PMA) was added to enhance macrophage differentiation. PMA treatment, which activates protein kinase C, induces a greater degree of differentiation in U937 cells as reflected by increased adherence and expression of surface markers associated with macrophage differentiation. In control wells, no PMA solution was added.

Analysis of the inhibition of macrophage differentiation was carried out as follows: PMA treatment was perfomed for 24 hours in presence of hUCESCs-CM, ASCs-CM (StemPro^®^, Invitrogen) or control medium.

In addition, the capacity of hUCESCs-CM to revert macrophage differentiation was analyzed. For this, PMA treatment was performed for 24 hours. Then, medium was removed, cells were washed with PBS, and hUCESCs-CM, ASCs-CM (StemPro^®^, Invitrogen) or control medium was added for another 24 hours.

In both tests, supernatant was removed and adherent cells were washed, trypsinized and collected. Cells were centrifuged for 5 min at 400g and resuspended in 100 μl of PBS. Differentiation to macrophages was monitored by the expression of monocyte differentiation marker CD11b using flow cytometry analysis. Cells were stained with PE-CD11b monoclonal antibody (BD Biosciences) and with 7-AAD to assess cell viability.

### Human Cytokine Antibody Array

Forty eight hours-conditioned medium from hUCESCs (fresh and lyophilized) and ASCs was subjected to profiling using RayBio®Human Cytokine Antibody Array G-Series 2000 that simultaneously detects 174 human cytokines. Culture medium without FBS was run in parallel as negative control. Signal intensity values representing detected cytokines were subtracted from the background and normalized to positive controls on the same membrane. Experimental steps and analyses were conducted according to the manufacturer's instructions. Signal intensity values of each cytokine assessed in hUCESCs-CM (fresh and lyophilized), ASCs-CM, and control medium are presented as mean ± SD.

### Statistical Analysis

Each experiment was performed at least 3 times. Values are expressed as mean ± standard deviation. Means were compared using 2-tailed Student's *t* test or 1-way ANOVA, with the Tukey-Kramer multiple comparison test for post-hoc comparisons. For overall survival analysis, Cox's univariate method was used. P values less than 0.05 were considered statistically significant. The SPSS 17.0 program (SPSS Inc.) was used for all calculations.

## SUPPLEMENTARY MATERIAL FIGURES AND TABLE


