# Supplementary material for: Potential therapeutic effect of the secretome from human uterine cervical stem cells against both cancer and stromal cells compared with adipose tissue stem cells
Source: Oncotarget. 2014 Sep 26;5(21):10692–708. doi: 10.18632/oncotarget.2530 (PMC4279403; doi:10.18632/oncotarget.2530)
Supplement: Supplementary file 1 [file oncotarget-05-10692-s001.pdf]

Potential therapeutic effect of the secretome from human uterine cervical stem cells against both cancer and stromal cells compared with adipose tissue stem cells

Supplementary Material

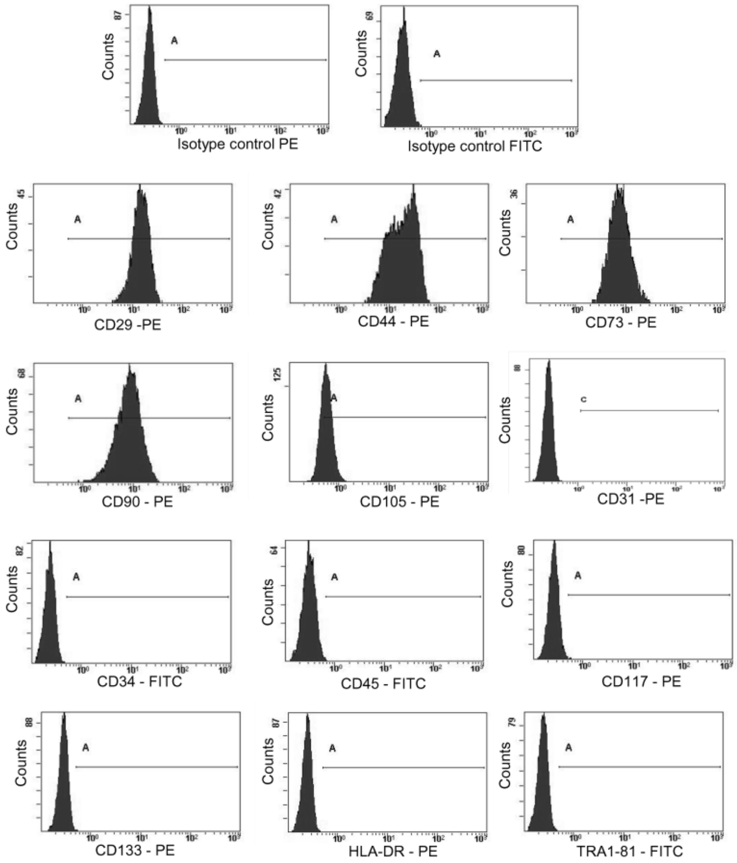

Supplemental figure 1: Flow cytometric analysis of hUCESCs. Representative figure of hUCESCs labeled with FITC- and PE- antibodies and examined by flow cytometry. Histograms showed the expression of surface antigens.

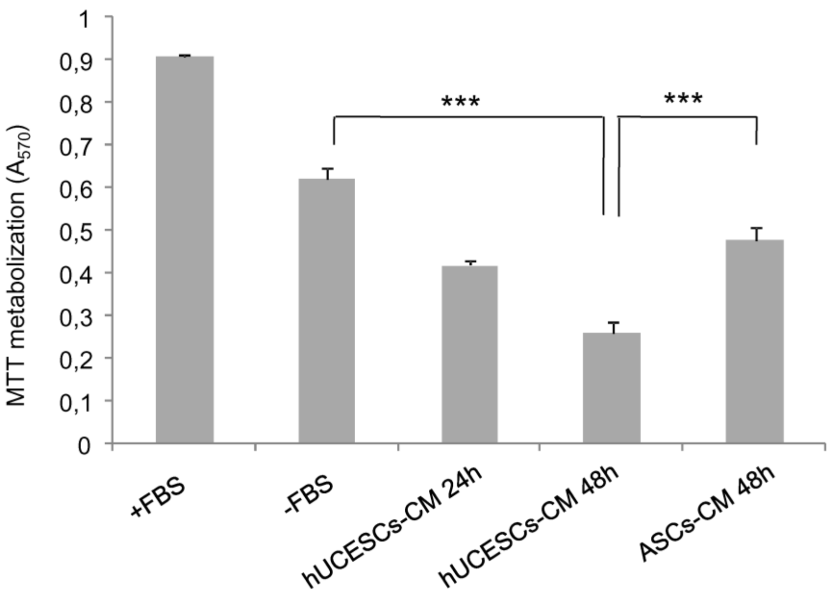

Supplemental figure 2: hUCESCs-CM reduces cell proliferation of the human cervical cancer HeLa cells. Cell proliferation assay of HeLa cells treated for 48 hours with complete medium (+FBS), incomplete medium (-FBS), 24 and 48-h hUCESCs-CM and 48-h ASCs-CM.

Supplemental table 1: Antibodies used.

Antigen	Source	Application
Actin HHF35	Dako	ICC
Active caspase-3 (asp175)	Cell Signaling	IHC, WB
Bid	Cell Signaling	WB
Bim (clone C3C5)	Cell Signaling	WB
Caspase 12	Cell Signaling	WB
Caspase 8 (D391)	Cell Signaling	WB
Caspase 9 (clone C9)	Cell Signaling	WB
CD90 (clon AS02)	Dianova	FC
CK (clone AE1/AE3)	Dako	ICC
Cleaved PARP	Cell Signaling	WB
Cyclin A	BD Biosciences	WB
Cyclin B	Santa Cruz Biotech	WB
Cyclin D1 (clone 7213G)	Santa Cruz Biotech	WB
Cyclin E	Santa Cruz Biotech	WB
Desmin	Dako	ICC
E-Cadherin (clone NCH-38)	Dako	ICC
Fibroblast marker	Santa Cruz Biotech	ICC
GAPDH	Santa Cruz Biotech	WB
KLF4 (clone B-9)	Santa Cruz Biotech	ICC
OCT4 (clone 7F9.2)	Millipore	ICC
Smooth muscle actin	Dako	ICC
Sox2 (clone SOX2-6)	Sigma-Aldrich	ICC
Vimentin (clone V9)	Dako	ICC
β-catenin (clone 1)	Dako	ICC

ICC: immunocytochemisry IHC: immunohistochemistry; WB: Western blot; FC: Flow cytometry.
